# Transposon engineering of *Saccharomyces cerevisiae* for allantoin-inducible cordycepin production

**DOI:** 10.48130/els-0026-0009

**Published:** 2026-08-24

**Authors:** Chaoqun Tang, Junyi Wang, Xueyi Luo, Haofeng Chen, Yang Zhang, Jifeng Yuan

**Affiliations:** 1 State Key Laboratory of Cellular Stress Biology, School of Life Sciences, Faculty of Medicine and Life Sciences, Xiamen, Xiamen University, Fujian 361102, China; 2 Department of Basic Medical Sciences, Qinghai University Medical College, Xining, Qinghai 810016, China; 3 School of Advanced Interdisciplinary Biomedical Sciences, Faculty of Medicine and Life Sciences, Xiamen University, Xiamen, Fujian 361102, China

**Keywords:** Cordycepin, Allantoin-inducible system, Transposon, Combinatorial optimization, *Saccharomyces cerevisiae*

## Abstract

Cordycepin is a high-value bioactive compound with significant therapeutic potential. In this study, we report a transposon-based genome engineering strategy for establishing an allantoin-inducible cordycepin production platform in *Saccharomyces cerevisiae*. We first optimized the allantoin-inducible system by refining the culture medium's components to improve cell growth and achieved an approximately 50-fold on/off ratio after reaching a plateau, thereby enhancing the robustness of engineered yeast strains. Next, a multiselection marker transposon system was implemented to achieve combinatorial integration of cordycepin biosynthetic genes (*cns1*/*cns2*) into the yeast genome. Engineered yeast strains obtained from the combinatorial library displayed varying cordycepin titers. Among them, one engineered strain produced up to 135 mg/L cordycepin, which is significantly higher than that achieved by the episomal plasmid-based strain. This work demonstrated a transposon-based workflow for combinatorial pathway optimization, which will be of particular interest for future microbial cell factory design.

## Introduction

Cordycepin (3′-deoxyadenosine) is a naturally occurring adenosine analog originally isolated from *Cordyceps* species and has attracted considerable attention because of its broad spectrum of biological activities, including antitumor, anti-inflammatory, immunomodulatory, and metabolic regulatory effects^[1−3]^. Its unique 3′-deoxyribose structure enables cordycepin to interfere with RNA synthesis, polyadenylation, and multiple adenosine-dependent signaling processes^[4]^, making it an important bioactive molecule for pharmaceutical development and manufacturing functional products. However, the commercial supply of cordycepin still relies largely on extraction from cultivated *Cordyceps* biomass or fermentation with naturally producing fungi. These production processes are constrained by slow fungal growth, complex culture conditions, batch-to-batch variability, relatively low productivity, and difficult downstream purification. Chemical synthesis provides an alternative route but generally requires multistep reactions and extensive protection and deprotection of functional groups, limiting its economic and environmental sustainability. Therefore, the development of efficient microbial cell factories for the sustainable biosynthesis of cordycepin has become an important objective in metabolic engineering and industrial biotechnology.

The identification of the cordycepin biosynthetic gene cluster in *Cordyceps militaris* has provided a genetic basis for reconstructing cordycepin biosynthesis in heterologous microbial hosts. In particular, the Cns1- and Cns2-mediated biosynthetic pathway has been introduced into several microbial platforms to enable heterologous *de novo* cordycepin production. Compared with native fungal fermentation, engineered microorganisms offer the advantages of genetic tractability, cultivation efficiency, control over the process, and pathway optimization. Among heterologous hosts, *Saccharomyces cerevisiae* has attracted increasing interest for cordycepin production. Early studies demonstrated that heterologous expression of *cns1* and *cns2* enabled cordycepin biosynthesis in *S. cerevisiae*, establishing the feasibility of reconstructing the fungal pathway in yeast^[5]^. Subsequent studies improved cordycepin production through fermentation optimization and systematic metabolic engineering strategies, including enhancement of the precursor supply, rewiring of the purine metabolism, modulation of competing pathways, and optimization of cellular energy and cofactor availability^[6−10]^. Systematic chassis selection combined with extensive metabolic engineering and fed-batch fermentation has further increased cordycepin production to grams per liter levels^[8]^, highlighting the considerable potential of *S. cerevisiae* as an industrial host for cordycepin biosynthesis.

However, traditional engineering of *S. cerevisiae* relies on plasmid-based expression systems or targeted integration into specific genetic loci. Plasmid-based approaches enable rapid pathway construction but often suffer from segregational instability, heterogeneous copy number distribution, and dependence on continuous selection pressure, whereas targeted chromosomal integration requires the construction of locus-specific donor DNA and repeated genome engineering procedures. Multicopy integration strategies targeting repetitive genomic elements, including *δ*-sequences, rDNA repeats, and CUP1 loci, have been developed to simultaneously introduce multiple copies of heterologous pathways into the yeast genome to facilitate combinatorial pathway assembly and gene dosage optimization^[11−18]^. DNA transposon systems provide another powerful strategy for stable genomic engineering by enabling the efficient mobilization and integration of heterologous DNA cargos^[19,20]^. For instance, the piggyBac transposon system mediates efficient cut-and-paste transposition of DNA cargoes flanked by terminal repeat sequences into TTAA target sites and has been developed as a versatile tool for genome engineering in eukaryotic microorganisms^[21]^, including *S. cerevisiae* and *Komagataella phaffii* (previously designated as *Pichia pastoris*). Nevertheless, the application of piggyBac for the combinatorial integration of metabolic pathways and systematic gene dosage engineering remains largely unexplored.

**Figure 1:**
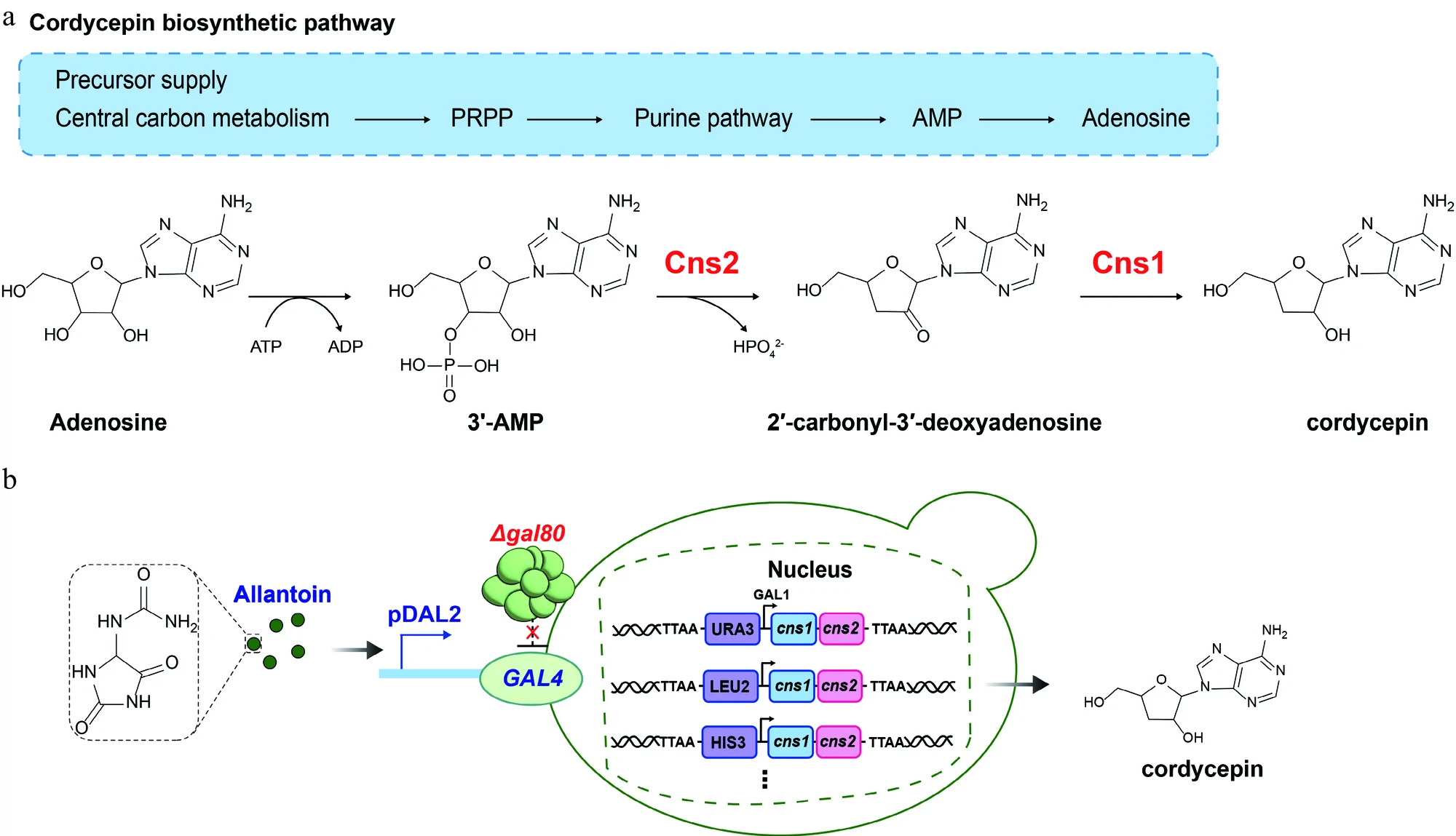
Combinatorial integration of the cordycepin biosynthetic pathway for enhanced production in *S. cerevisiae*. (a) Schematic diagram of the cordycepin biosynthesis pathway. Phosphoribosyl pyrophosphate, PRPP; adenosine 5'-monophosphate, 5'-AMP, AMP; adenosine 3'-monophosphate, 3'-AMP. (b) Schematic illustration of multimarker combinatorial integration of the *cns1*/*cns2* expression cassette. Cns1 and Cns2 were connected by a self-cleavable P2A peptide, enabling dual gene expression controlled by a single galactose-inducible promoter (GAL1) promoter.

Here, we report a piggyBac-mediated combinatorial genomic integration strategy for pathway engineering in *S. cerevisiae* toward high-level cordycepin production (Fig. 1). We first optimized our previously established allantoin-inducible system by modifying the culture medium's composition, improving cellular growth while maintaining stringent transcriptional control with an approximately 50-fold on/off expression ratio. Subsequently, we used the piggyBac transposon system to combinatorially integrate the cordycepin biosynthetic pathway genes (*cns1* and *cns2*) into the yeast genome. Engineered strains generated from the combinatorial integration library exhibited diverse levels of cordycepin. Among them, the highest-producing strain achieved a cordycepin titer of 135 mg/L in a shaker tube, which was substantially higher than that achieved by the episomal plasmid-based strain. Overall, this work provides a readily applicable genetic toolbox for combinatorial pathway optimization in yeast, which can be further expanded for the construction of complex multigene biological pathways.

## Materials and methods

### Strains, media, and culture conditions

*S. cerevisiae* JS-Allan^[22]^ was used as the parental strain. The parental strain was cultivated in a yeast peptone dextrose (YPD) medium. All strains were cultured at 30 °C with shaking at 250 r/min. Small-scale fermentation was performed in a 2-mL culture volume. Genomic integration strains were cultured in YNBD, YABD, YNBD (−DO), or YNAD (−DO) media, whereas the plasmid-based strain was cultured in YABD medium without uracil supplementation. The YNBD medium was used as a chemically defined medium and consisted of a yeast nitrogen base (YNB) without amino acids and with a synthetic drop-out amino acid mixture of ammonium, glucose, and yeast (DO, Sigma-Aldrich, Cat. no. Y1501), with 5.0 g/L ammonium sulfate as the nitrogen source. The YABD medium contained the same components as the YNBD medium, except that ammonium sulfate was replaced with 1.7 g/L of allantoin as the nitrogen source. YNBD (−DO) and YABD (−DO) media were prepared by omitting the DO supplement from the YNBD and YABD media, respectively. The chemically competent *Escherichia coli* TOP10 (purchased from VEIDI Biologicals Company) was cultured at 37 °C in Luria–Bertani medium (10 g/L tryptone, 5 g/L yeast extract, and 10 g/L NaCl) and used as the host strain for plasmid propagation, cloning, and selection under ampicillin pressure. All restriction enzymes, T4 ligase and high-fidelity Phusion polymerase were all purchased from New England Biolabs (Ipswich, USA).

### Plasmid construction and genome editing

For constructing enhanced green fluorencent protein (EGFP) reporter system, the plasmid pUC18PB-URA3-GAL1-EGFP was constructed from pUC18BP-URA3-TEF2-EGFP^[20]^ by replacing the TEF2 promoter with the galactose-inducible promoter (GAL1) promoter through *Sac*I and *Bam*HI digestion and ligation. The plasmids pUC18PB-LEU2-GAL1-EGFP and pUC18PB-HIS3-GAL1-EGFP were constructed using pRS413GAL1-EGFP and pRS415GAL1-EGFP as templates. In brief, DNA fragments containing the corresponding auxotrophic selection markers and the GAL1p-EGFP-CYC1t expression cassette were amplified using the primer pair pGAL1_BglII_fwd and marker_SalI_rev. The amplified fragments were digested with *Bgl*II and *Sal*I and ligated into the pUC18 backbone^[20]^ digested with *Bam*HI and *Sal*I, generating the corresponding marker-containing reporter plasmids. The cordycepin biosynthetic expression plasmid pRS426GAL1-cns1-cns2 was generated from pRS426GAL1-EGFP by replacing the EGFP sequence with the codon-optimized cns1-P2A-cns2 module through *Bam*HI and *Xho*I digestion and ligation. For construction of the piggyBac donor plasmids, the marker-containing pUC18PB backbones (URA3, LEU2, and HIS3) were linearized by *Bam*HI and *Xho*I digestion and assembled with the *cns1*–*cns2* expression cassette (*Bam*HI–*Xho*I) using T4 DNA ligase. The resulting piggyBac donor plasmids were pUC18PB-URA3-GAL1-cns1-cns2, pUC18PB-LEU2-GAL1-cns1-cns2, and pUC18PB-HIS3-GAL1-cns1-cns2. All plasmids and oligonucleotides used in this study are listed in Table 1.

**Table 1:** List of plasmids and oligonucleotides used in this study.

Name	Description	Source
p414TEF2-PBase	Plasmid carrying a TRP1 marker and a TEF2 promoter-driven PBase	[20]
pUC18BP-URA3-TEF2-EGFP	A transposon plasmid carrying the URA3 selection marker, the TEF2 promoter, the *EGFP* reporter gene, and inverted terminal repeats (ITRs).	[20]
pUC18PB-URA3-GAL1-EGFP	pUC18BP-URA3-TEF2-EGFP derivative replacing the TEF2 promoter with the GAL1 promoter.	This study
pUC18PB-LEU2-GAL1-EGFP	pUC18PB-URA3-GAL1-EGFP derivative replacing URA3 selection marker with LEU2	This study
pUC18PB-HIS3-GAL1-EGFP	pUC18PB-URA3-GAL1-EGFP derivative replacing URA3 selection marker with HIS3	This study
pUC57-kan-cns1-cns2	Plasmid pUC57-kan derivative with synthesized *cns1*-P2A-*cns2*	This study
pRS426GAL1-cns1-cns2	Plasmid pRS426GAL1 carrying a URA3 marker and a GAL1 promoter-driven *cns1*-P2A-*cns2*	This study
pUC18PB-URA3-GAL1-cns1-cns2	pUC18PB-URA3-GAL1-EGFP derivative replacing the EGFP gene with the *cns1*–P2A–*cns2* expression cassette	This study
pUC18PB-LEU2-GAL1-cns1-cns2	pUC18PB-LEU2-GAL1-EGFP derivative replacing the EGFP gene with the *cns1*–P2A–*cns2* expression cassette	This study
pUC18PB-HIS3-GAL1-cns1-cns2	pUC18PB-HIS3-GAL1-EGFP derivative replacing the EGFP gene with the *cns1*–P2A–*cns2* expression cassette	This study
pGAL1_BglII_fwd	TTGGTCTCAGATCTACCCTCACTAAAGGGAAC	This study
marker_SalI_rev	AGCGCGTCGACCATGCAGCTCCCGGAGACG	This study
pUC18PB_SalI_fwd	AGCGCGTCGACCATGCGTCAATTTTACGCATGATTATCTTTAACGTACGTCACAATATGATTATCTTTCTAGGGCTAATGAGTGAGCTAACTCAC	This study
pUC18PB_BamHI_rev	CGCGGATCCCATGCGTCAATTTTACGCAGACTATCTTTCTAGGGTGGCGAATGGCGCCTGATGC	This study

The piggyBac donor plasmids were co-transformed into JS-Allan competent cells together with the piggyBac transposase expression plasmid p414TEF2-PBase^[20]^. Transformants were selected on synthetic dropout media lacking the corresponding auxotrophic supplements to obtain genomic integrants. Integration of the pUC18PB-URA3-GAL1-EGFP donor cassette generated the reporter strain JSA-URA-EGFP. Integration of the pUC18PB-URA3-GAL1-cns1-cns2 donor cassette generated the single-marker cordycepin-producing strains JSA-COR-S1~S20. To increase pathway dosage, a combinatorial piggyBac integration strategy was applied, using multiple auxotrophic markers (URA3, LEU2, and HIS3). Multiple GAL1-driven *cns1*–*cns2* expression cassettes carrying different selection markers were integrated into the JS-Allan genome, generating the multimarker genomic integrants JSA-COR-M1~M20. The plasmid-based cordycepin-producing strain harboring pRS426GAL1-cns1-cns2 was designated JSA-p426-COR. All yeast strains used in this study are listed in Table 2. After genomic integration and plasmid curing under nonselective conditions, individual colonies were screened by polymerase chain reaction (PCR) for the presence of the piggyBac transposase (PBase). Only PBase-negative colonies were selected for the subsequent fermentation experiments.

**Table 2:** List of strains used in this study.

Strains	Description	Source
JS-Allan	BY4741 Δgal80 pDAL2::GAL4	[22]
JSA-p426-COR	JS-Allan derivative transformed with pRS426GAL1-cns1-cns2	This study
JSA-URA-EGFP	These strains represent independent single URA3-marker integrants harboring a GAL1-EGFP expression cassette	This study
JSA-COR-S1~S20	JS-Allan derivative with single-marker integration of URA3-labeled GAL1-*cns1*–*cns2* expression cassettes	This study
JSA-COR-M1~M20	JS-Allan derivative with multimarker integration of URA3-, LEU2-, and HIS3-labeled GAL1-*cns1*–*cns2* expression cassettes	This study

### Fluorescence and metabolite analysis

To evaluate the impact of different nitrogen sources on the expression performance of the allantoin-responsive galactose-inducible promoter (GAL) system, the single-marker genomic integrant JSA-URA-EGFP was cultivated under three nutritional conditions with DO supplementation: The YABD medium containing allantoin as the inducing nitrogen source, the conventional YNBD medium containing ammonium as the preferred nitrogen source, and a mixed nitrogen condition consisting of 10% YNBD and 90% YABD. In this analysis, 100 μL of the cell culture was taken for measuring optical density at 600 nm (OD_600_) and EGFP signals with a microplate reader (Biotek, Synergy H1). EGFP expression was monitored over a 120-h cultivation period by measuring fluorescence intensity and normalizing the values to cell density (EGFP/OD_600_). Fluorescence measurements were performed using a microplate reader (excitation wavelength: 480 nm; emission wavelength: 520 nm). The on/off expression ratio was calculated as the normalized EGFP fluorescence under inducing conditions (YABD medium or 10% YNBD + 90% YABD medium) relative to the noninducing condition (YNBD medium).

Cordycepin concentrations were determined by the Shimadzu Prominence LC-20A system equipped with a photodiode array detector and a reversed-phase C18 column (4.6 mm × 250 mm, 5-μm particle size). The mobile phase consisted of Solvent A (deionized water containing 0.1% trifluoroacetic acid, TFA) and Solvent B (acetonitrile containing 0.1% TFA) with ultraviolet (UV) detection at 260 nm. Isocratic elution was performed using a mobile phase consisting of 92.5% Solvent A and 7.5% Solvent B (v/v). Chromatographic separation was performed at a flow rate of 1 mL/min with an injection volume of 10 μL. Cordycepin was identified by comparing the retention time of fermentation samples with that of an authentic cordycepin standard, which exhibited a retention time of approximately 3 min under the described chromatographic conditions. Quantification was performed using a calibration curve generated from serial dilutions of the cordycepin standard (25, 50, 100, and 200 mg/L). The calibration curve was generated by plotting the peak area against the corresponding cordycepin concentration. All fermentation samples were collected after 96 h of cultivation and filtered before subjected to high-performance liquid chromatography (HPLC) analysis.

## Results

### Optimization of the culture medium's components for improved allantoin-inducible GAL system

**Figure 2:**
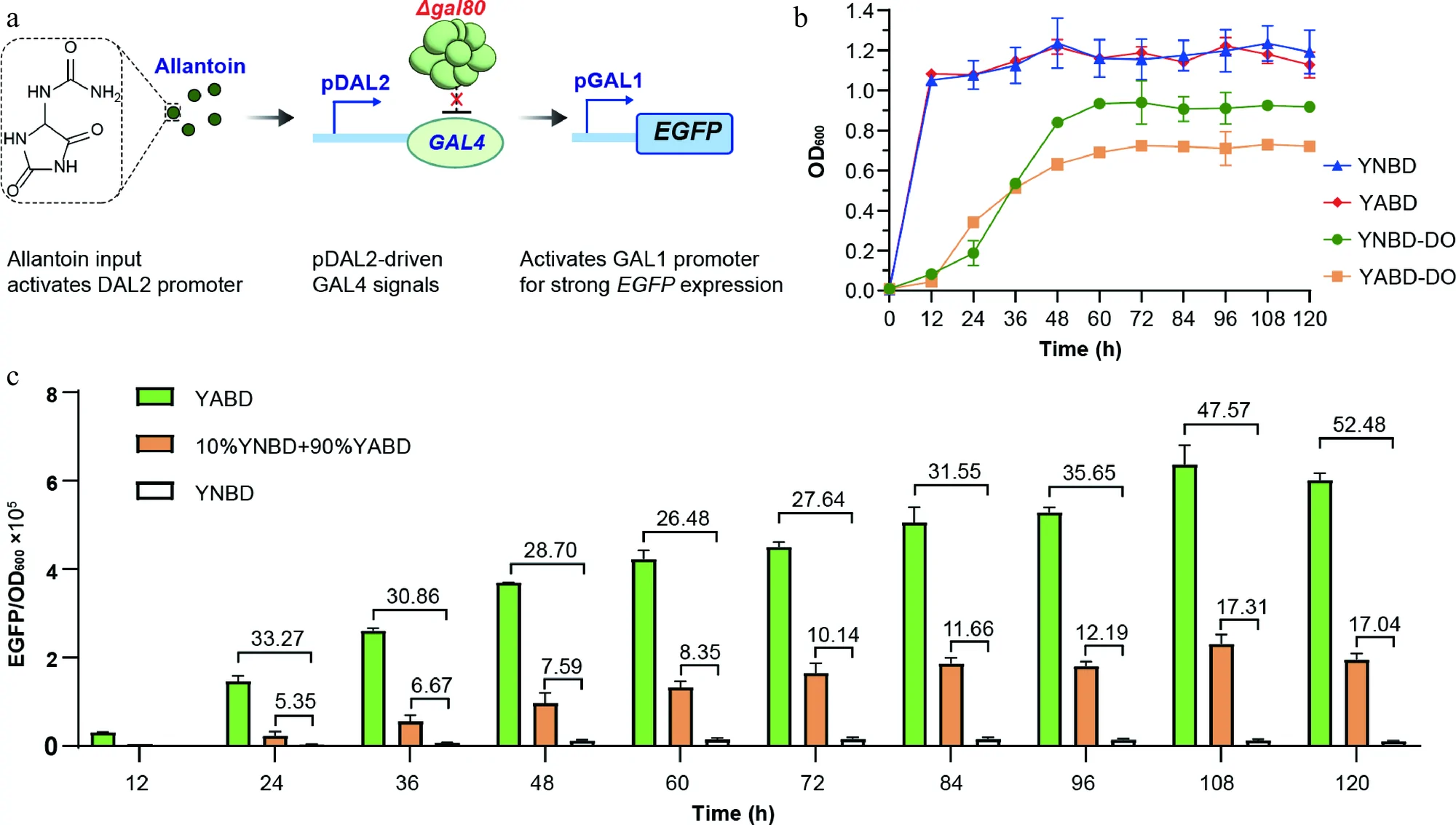
Allantoin–GAL system for controlling EGFP expression in *S. cerevisiae*. (a) Schematic illustration of allantoin-inducible *EGFP* expression in yeast. (b) Growth comparison of JSA-URA-EGFP under different nutrient conditions. The growth curves of JSA-URA-EGFP were obtained in DO-supplemented YNBD medium (blue line), DO-supplemented YABD medium (red line), DO-free YNBD medium (green line) and DO-free YABD medium (orange line). (c) *EGFP* expression regulated by the allantoin-responsive GAL system in the JSA-URA-EGFP strain. The strain JSA-URA-EGFP was cultivated in YNBD, YABD, and a mixture of 10%YNBD + 90%YABD, and 100 μL of the cell culture was taken for measuring OD_600_ and EGFP signals with a microplate reader (Biotek, Synergy H1). All experiments were conducted in triplicate, and data represent the mean values with standard deviations. Statistical significance was determined using one-way analysis of variance (ANOVA) followed by Tukey's multiple comparisons test.

Traditional gene expression in yeast primarily relies on constitutive promoters (e.g., PGK1, TEF1) or galactose-inducible promoters (GAL1/10)^[23]^. However, constitutive promoters often impose a metabolic burden, whereas carbon source-dependent inducible systems are inhibited under glucose repression, limiting their utility for industrial-scale applications^[24]^. To overcome these limitations, layered genetic circuit designs have been developed to amplify weak environmental inputs into robust transcriptional outputs^[25,26]^, and have been demonstrated in cyanamide- and copper-inducible GAL systems^[27,28]^, which utilize signal cascade architectures to achieve enhanced gene expression. Previously, we developed a layered allantoin–GAL regulatory system in which the allantoin-responsive DAL2 promoter drives GAL4's expression, creating a transcriptional amplification cascade that activates GAL1-dependent downstream gene expression^[22]^ (Fig. 2a). This system converts extracellular allantoin signals into amplified transcriptional outputs and achieved a 68.6-fold induction under inducing conditions. This nutrient-responsive architecture provides an intrinsic regulatory mechanism that couples metabolic output with environmental signals and minimizes unintended expression under noninducing conditions. However, removal of essential nutrient supplements during selective cultivation substantially impaired cellular growth^[22]^, limiting this system for practical metabolic engineering applications.

As shown in Fig. 2b, consistent with our previous observations^[22]^, the strain JSA-URA-EGFP, derived from JS-Allan through piggyBac-mediated integration of the GAL1-EGFP expression cassette, exhibited reduced biomass accumulation upon the addition of allantoin in DO-deficient conditions. Notably, upon DO supplementation, JSA-URA-EGFP maintained comparable growth profiles in YABD (red line) and YNBD (blue line), reaching a stable biomass level of OD_600_ ~1.2 (Fig. 2b). Further characterization of EGFP expression revealed that the strain JSA-URA-EGFP exhibited a strong and sustained EGFP induction profile in the YABD medium (Fig. 2c), suggesting that DO supplementation alleviated nutritional limitations without interfering with allantoin-dependent activation of the GAL circuit. EGFP/OD_600_ gradually increased throughout cultivation and reached a maximum level of approximately 6.3 × 10^5^ at 108 h. In contrast, EGFP expression remained consistently low under YNBD conditions as expected, owing to ammonium-mediated repression of the DAL signal pathway. The on/off ratio between YABD and YNBD was approximately 50-fold after reaching a plateau, which is similar to that of the single-copy plasmid-based EGFP expression system (67.2-fold)^[22]^.

Moreover, we also investigated the strain performance in the mixed nitrogen source condition (10% YNBD + 90% YABD), and the strain JSA-URA-EGFP displayed a moderate expression profile between fully induced and repressed states. The tunable response under mixed nitrogen conditions further highlights the potential for future balancing of expression strength with improved host physiological fitness and regulatory precision. Collectively, these findings highlight that culture medium optimization is a critical step for translating the allantoin-responsive expression system from a proof-of-concept demonstration toward practical metabolic engineering applications.

### PiggyBac-mediated combinatorial integration of the cordycepin biosynthetic pathway

In the cordycepin biosynthetic route, *cns2* catalyzes the conversion of adenosine 3'-monophosphate (3'-AMP) into the key intermediate 2'-carbonyl-3'-deoxyadenosine (2'-C-3'-dA), which is subsequently reduced by *cns1* to generate cordycepin (Fig. 1). This two-step enzymatic cascade represents the core biosynthetic module responsible for heterologous cordycepin production. On the basis of this pathway architecture, we selected the *cns1*/*cns2* gene pair as the minimal and functional biosynthetic cassette and placed it under the control of the allantoin-responsive GAL regulatory system, enabling nutrient-dependent activation of cordycepin biosynthesis.

**Figure 3:**
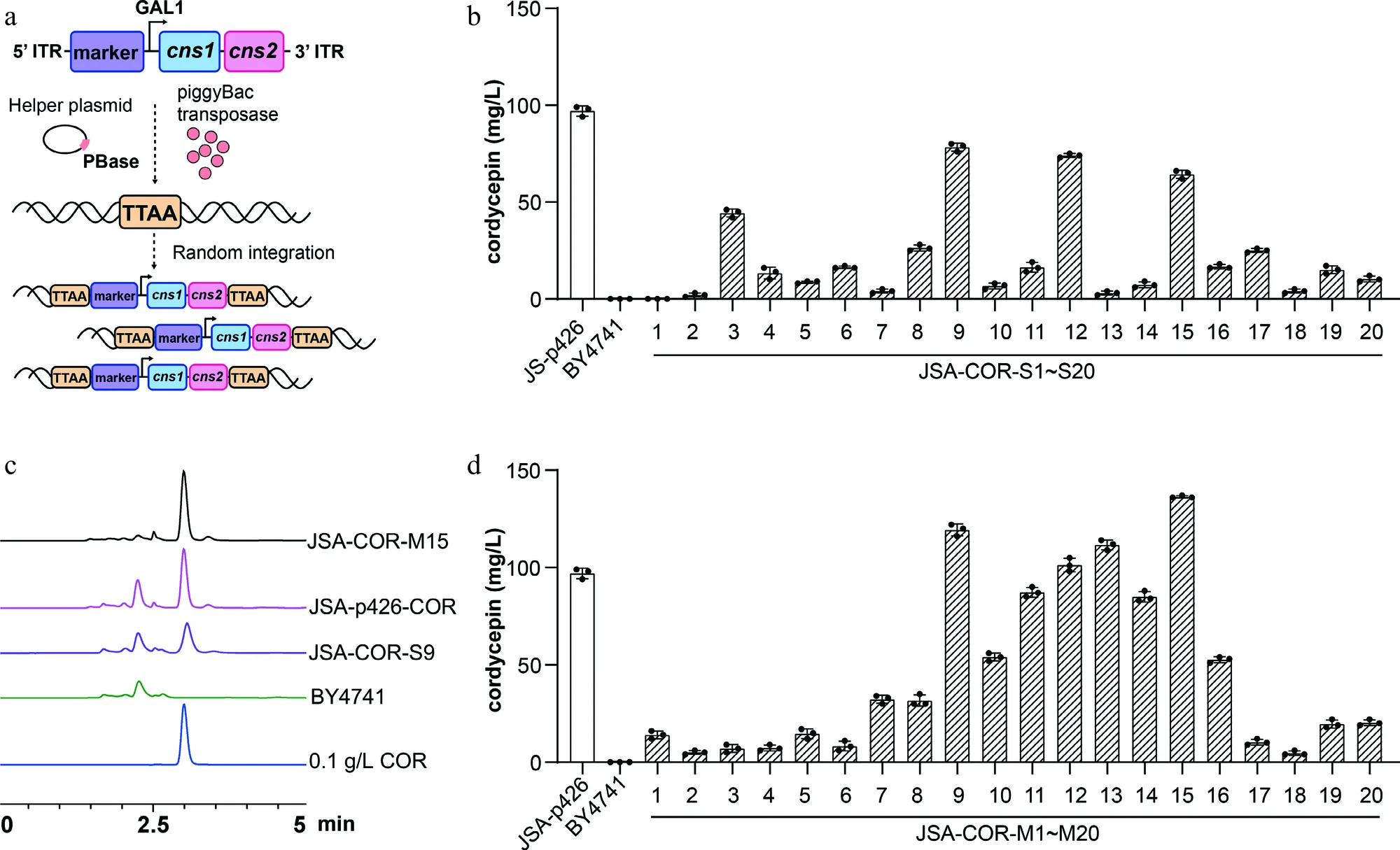
Characterization of combinatorial library for cordycepin biosynthesis in *S. cerevisiae*. (a) Schematic illustration of combinatorial piggyBac transposon-mediated integration of the *cns1*/*cns2* expression cassette using multiple auxotrophic selection markers. (b) Comparative analysis of cordycepin production in 20 strains (JSA-COR-S1~S20) constructed via single-marker piggyBac transposon integration. (c) Representative HPLC results of cordycepin production in engineered strains. (d) Comparative analysis of cordycepin production in 20 strains (JSA-COR-M1~M20) constructed via multimarker integration of the piggyBac transposon. The strain JSA-p426-COR indicates the multicopy episomal plasmid-based engineered strain used for cordycepin biosynthesis. Cordycepin production was screened under a 2-mL fermentation condition. All experiments were conducted in triplicate, and data represent the mean values with standard deviations.

The piggyBac donor construct was generated by flanking the GAL1-cns1-cns2 expression cassette with the piggyBac terminal repeat sequences PB5 and PB3. Upon co-expression of the piggyBac transposase (PBase) (Fig. 3a), the donor cassette, together with the selection marker, was mobilized and integrated into genomic TTAA target sites, generating a genomic integrant library. This design enabled chromosomal incorporation of the cordycepin biosynthetic module while reducing the genetic instability commonly associated with episomal plasmid-based expression systems. Independent single-marker integrants (JSA-COR-S1~S20) produced varying levels of cordycepin (Fig. 3b). Quantitative analysis of multiple independent transformants further revealed variability in cordycepin production, with the highest-producing transformant achieved a cordycepin titer of 78 mg/L in a shaker tube. HPLC analysis revealed the distinct cordycepin peak in all engineered strains, whereas no corresponding product was detected in the control strain, indicating that the heterologously expressed Cns1 and Cns2 enzymes were functionally active in *S. cerevisiae* (Fig. 3c).

To further increase pathway dosage, we used a combinatorial piggyBac integration strategy using multiple auxotrophic markers (URA3, LEU2, and HIS3), generating the genomic integrants JSA-COR-M1~M20. Independent donor constructs carrying identical GAL1-driven *cns1*–*cns2* expression cassettes but different selection markers were introduced into the JS-Allan (JSA) genome, enabling the simultaneous incorporation of multiple copies of the biosynthetic module. Through this multimarker integration strategy, engineered strains carrying multiple chromosomal GAL1-cns1-cns2 expression units were generated, providing a genomic integration platform for combinatorial pathway optimization. As shown in Fig. 3d, the majority of combinatorial integration strains exhibited higher cordycepin titers than the single URA3-marker integration strains. Notably, the highest-producing strain achieved a cordycepin titer of 135 mg/L in a shaker tube, representing an approximately 1.73-fold increase compared with the best-performing JSA-COR-S9 strain. Although variation in production levels was observed among individual transformants, combinatorial integration consistently improved the overall cordycepin titer, demonstrating that piggyBac-mediated multimarker combinatorial integration is an effective strategy for increasing pathway dosage.

## Discussion

In this study, the piggyBac transposon system was used to integrate the EGFP reporter and the cordycepin biosynthetic genes *cns1*/*cns2* into the yeast genome. Compared with episomal plasmid-based expression systems, this strategy not only enhances genetic stability but also minimizes plasmid-associated dissemination of engineered genetic elements^[29]^. Small-scale cultivation results showed that this transposon-based engineering system achieved substantially enhanced metabolic production, with cordycepin titer reaching 78 mg/L after 96 h of cultivation in YABD medium. Additionally, a multimarker integration strategy was applied. On the basis of the small-scale screening results, the cordycepin titer of the highest-producing strain was 135 mg/L, representing a 1.73-fold increase compared with that of the best performing single-marker genomic integrant. Considering that allantoin is not a predominant nitrogen source in natural environments, the designed allantoin-inducible system may provide a potential biocontainment advantage by restricting cordycepin production under environmental conditions lacking allantoin, similar to the biocontainment feature achieved by rational genetic circuit design^[30,31]^. However, the current study primarily demonstrates the nutrient-responsive regulatory capability of this system, and its actual effectiveness for environmental containment requires further experimental validation.

Although the cordycepin production level obtained in this study remains below industrial-scale production levels, optimized fermentation and bioprocess engineering have been demonstrated to substantially enhance cordycepin production. Cordycepin biosynthesis is highly sensitive to the gene dosage and regulatory architecture. Metabolic pathway optimization based on systems biology, together with synthetic biology approaches (including promoter engineering and copy number regulation), has enabled cordycepin production exceeding 1 g/L in a 5-L bioreactor^[7−9,32,33]^, indicating that the allantoin-inducible cordycepin-producing strain constructed in this study holds considerable potential for further optimizing fermentation and scaled-up production. However, precise characterization of the genomic architecture of the engineered strains remains necessary to better understand the relationship between genomic integration patterns and production performance. Future studies will require further characterization of these strains using approaches such as whole-genome sequencing, integration site mapping, and quantitative copy number analysis to precisely resolve their genomic structures. Future efforts coupled with high-throughput screening approaches are expected to select superior cordycepin-producing strains. Collectively, our work demonstrates a transposon-based workflow for combinatorial pathway optimization with improved chemical production, which will be broadly applicable to the combinatorial design of other microbial cell factories.

## Data Availability

The data that support the findings of this study are available from the corresponding author upon reasonable request.
